# P-87. Real-world Efficacy and Outcomes Implementing Oral Antibiotics for Treatment of Bone and Joint Infections

**DOI:** 10.1093/ofid/ofae631.294

**Published:** 2025-01-29

**Authors:** Marten R Hawkins, Elizabeth Thottacherry, Prerak Juthani, Jenny R Aronson, Amy Chang, Derek F Amanatullah, Anirudh Tarimala, Jessie T Markovits, Sa Shen, Marisa Holubar, Daisuke Furukawa

**Affiliations:** University of Michigan, Stanford, California; Stanford Health Care, Stanford, California; Stanford, Cupertino, California; Stanford University, Stanford, California; Stanford University, Stanford, California; Stanford University, Stanford, California; Stanford University, Stanford, California; Stanford University School of Medicine, Stanford, California; Quantitative Sciences Unit, Stanford, California; Stanford University School of Medicine, Stanford, California; Stanford University, Stanford, California

## Abstract

**Background:**

Traditionally, bone and joint infections have been treated with several weeks of intravenous (IV) antibiotic therapy; however clinical trial data support the safety and efficacy of oral antibiotics. Despite this, many physicians still prefer treating these infections with IV antibiotics, and studies on the real-world application of these clinical trials are limited.

Selected Sections from Stanford bone and joint infections oral treatment guide

**Figure:**
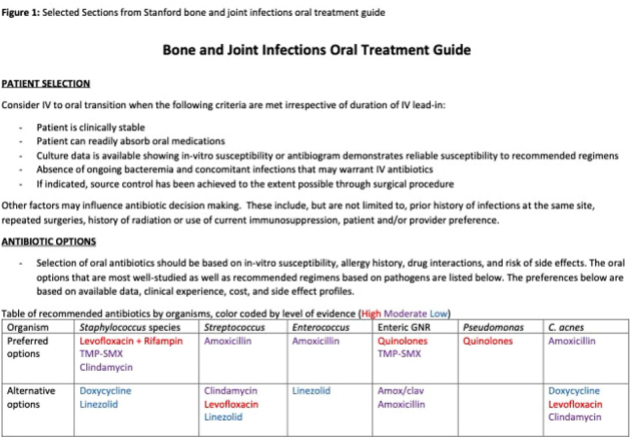

**Methods:**

In April 2023, we instituted a multi-disciplinary initiative to implement a new guideline (Figure 1) to preferentially treat bone and joint infections with oral antibiotics. Patients were included in our prospective analysis if they were hospitalized at Stanford with a bone or joint infection, >18 years of age and seen by the infectious diseases consult team. A baseline pre-implementation cohort of patients discharged between 4/1/2022 and 3/31/2023 was identified through billing codes and screened for the same inclusion criteria via manual chart review. Treatment failure was defined as clinical, microbiological or pathological evidence of failure within 3 months of discharge.

**Results:**

One-hundred eighty-seven patients (53 pre-implementation and 134 post-implementation) were included in the analysis. The most common infection type was prosthetic joint infection occurring in 70 (37%) followed by diabetic foot osteomyelitis in 41 (22%). In the pre-implementation cohort, 25% (13/53) of patients were discharged exclusively on oral antibiotics compared to 69% (93/134) in the post-implementation cohort (p< 0.01), with no difference in the percentage of patients meeting our criteria (Fig. 1) for oral antibiotics (70% vs 60%; p=0.23). There was no difference in the rate of treatment failure between cohorts (8% [4/53] vs. 9% [12/134]; p=0.76), but the median hospital length of stay was shorter in the post-implementation cohort (7 vs 8 days; p=0.04). The post-implementation cohort trended toward fewer central line-related adverse events (6% vs. 1% p=0.07) and increased discharge home (55% vs. 66%, p=0.14).

**Conclusion:**

An institutional guideline promoting oral antibiotics for bone and joint infections can be effectively implemented. This guideline led to similar clinical outcomes while reducing length of stay and may improve other patient-centered metrics.

**Disclosures:**

**Derek F. Amanatullah, MD/PhD**, Arthrology Consulting: Modular Cones, Mixed Reality, Acetabular Augments, Off-center Liners|Arthrology Consulting: Ownership Interest|DePuy: Advisor/Consultant|Knlmbe Designs: Knee Balancing|Knlmbe Designs: Ownership Interest|Medacta: Advisor/Consultant|nSight Surgical: Stocks/Bonds (Private Company)|PlantarTech: Dynamic Hallux Splinting|PlantarTech: Ownership Interest|QT Ultrasound: Stocks/Bonds (Private Company)|Recoup Fitness: Stocks/Bonds (Private Company)|Stanford: Retractor, Phage-hydrogel, Computer Vision|WedMD: Honoraria|Well Beam: Advisor/Consultant|Well Beam: Stocks/Bonds (Private Company)

